# Correction to: A new multiple trauma model of the mouse

**DOI:** 10.1186/s12891-018-2330-1

**Published:** 2019-02-11

**Authors:** Stefanie Fitschen-Oestern, Sebastian Lippross, Tim Klueter, Matthias Weuster, Deike Varoga, Mersedeh Tohidnezhad, Thomas Pufe, Stefan Rose-John, Hagen Andruszkow, Frank Hildebrand, Nadine Steubesand, Andreas Seekamp, Claudia Neunaber

**Affiliations:** 1 Department of Trauma Surgery, University Medical Center of Schleswig-Holstein, Campus Kiel, Kiel, Germany; 2 Department of Anatomy and Cell Biology, RWTH Aachen University, Wendlingweg 2, 52074 Aachen, Germany; 3 Department of Biochemistry, Medical Faculty, Olshausenstr. 40, 24098 Kiel, Germany; 4 Department of Trauma Surgery, RWTH Aachen University, Pauwelsstraße 30, 52074 Aachen, Germany; 5 Department of Trauma Surgery, Hannover Medical School, Hannover, Germany

## Correction

After publication of the original article [1], it was noticed that the following corrections needed to be implemented:

1. Claudia Neunaber belongs to the Trauma Department of the Hannover Medical School, Hannover, Germany and is in charge of the research department.
2. Some important publications describing animal models combining chest trauma and fractures of the long bones have not been cited in the original article and have been included. Sections of the background part that highlight the uniqueness of the presented combination of thorax trauma and femur fracture stabilized by intramedullary fixation are capable of being misunderstood. Therefore, the appropriate sections have been corrected in this Correction article below.

## Abstract

### Background

Blunt trauma is the most frequent mechanism of injury in multiple trauma, commonly resulting from road traffic collisions or falls. Two of the most frequent injuries in patients with multiple trauma are chest trauma and extremity fracture. Several trauma mouse models combine chest trauma and head injury, but only a few trauma models include the combination of chest trauma and long bone fracture.

Outcome is essentially determined by the combination of these injuries. In this study, we attempted to establish a reproducible novel multiple trauma model in mice that combines blunt trauma, major injuries and simple practicability.

### Methods

Ninety-six male C57BL/6 N mice (*n* = 8/group) were subjected to trauma for isolated femur fracture and a combination of femur fracture and chest injury. Serum samples of mice were obtained by heart puncture at defined time points of 0 h (hour), 6 h, 12 h, 24 h, 3 d (days), and 7 d.

### Results

A tendency toward reduced weight and temperature was observed at 24 h after chest trauma and femur fracture. Blood analyses revealed a decrease in hemoglobin during the first 24 h after trauma. Some animals were killed by heart puncture immediately after chest contusion; these animals showed the most severe lung contusion and hemorrhage. The extent of structural lung injury varied in different mice but was evident in all animals. Representative H&E-stained (Haematoxylin and Eosin-stained) paraffin lung sections of mice with multiple trauma revealed hemorrhage and an inflammatory immune response. Plasma samples of mice with chest trauma and femur fracture showed an up-regulation of IL-1β (Interleukin-1β), IL-6, IL-10, IL-12p70 and TNF-α (Tumor necrosis factor- α) compared with the control group. Mice with femur fracture and chest trauma showed a significant up-regulation of IL-6 compared to group with isolated femur fracture.

### Conclusions

The multiple trauma mouse model comprising chest trauma and femur fracture enables many analogies to clinical cases of multiple trauma in humans and demonstrates associated characteristic clinical and pathophysiological changes. This model is easy to perform, is economical and can be used for further research examining specific immunological questions.

## Background

Multiple trauma accounts for a significant proportion of deaths worldwide [2]. The most frequent injuries in trauma patients are chest trauma, extremity fractures and head injuries [3].

Blunt chest trauma can result in significant morbidity in injured patients, and both chest wall and intrathoracic visceral injuries can lead to life-threatening complications if not anticipated and treated [4]. Extremity fractures such as a femur fracture must be stabilized.

The time point of operative treatment is still controversially discussed, although most of the literature recommends early surgical stabilization of these fractures. Respiratory deterioration can be exacerbated by the presence of unstable long bone fractures.

Several trauma mouse models focus on blunt chest trauma and head injury, but the combination of chest trauma and long bone fracture is less presented in animal models [5, 6].

Trauma causes tissue damage, blood loss and activation of the immune system. The extent of the inflammatory immune response correlates with the degree of tissue damage [7], whereas extremity fractures in particular are generally accompanied by extensive soft tissue damage in multiple trauma patients [8]. In addition, extremity fractures are associated with an increased risk of complications [9], which might explain why damage control surgery is currently a point of interest [10]. Cytokines are important components of the immune response, and their release correlates with the degree of trauma depending on the extent of the associated injury [11]. Cytokines such as IL-6 serve as markers for the severity of trauma and early identification of high-risk patients for the development of posttraumatic MODS (multi organ dysfunction syndrome) [12].

Chest trauma is associated with a serious risk of posttraumatic complications, including hypoxia caused by lung contusion, blood loss, heart contusion, pericardial tamponade or sepsis due to esophageal or tracheal perforations. Pulmonary contusion is the most frequently diagnosed intrathoracic injury related to blunt chest trauma, affecting 17–25% of adult blunt chest trauma patients [13]. It is also an independent risk factor for the development of pneumonia, severe clinical acute lung injury (ALI) and acute respiratory distress syndrome (ARDS) [14]. Lung contusion affects approximately 17–25% of adult patients with blunt trauma and is the leading cause of death from blunt thoracic injury [14]. Patients with concurrent blunt chest trauma and long bone fractures have an especially higher incidence of pulmonary damage [15].

The molecular mechanisms of the immune response after multiple trauma are highly complex and not yet completely understood. Only a few murine chest trauma models have been established to date and there are even less models that combine in general a chest trauma with an isolated femur fracture [5, 6, 16–18].

We aimed to develop a standardized, reproducible, and clinically relevant multiple trauma mouse model of chest trauma [19] and femur fracture [20] to investigate the pathophysiologic changes, especially cytokine expression, after multiple trauma. Both methods have been evaluated in isolation in several studies and described as reproducible [21–25].

In addition to clinical parameters, we focused on posttraumatic cytokine release based on knowledge of the tight correlation between immunological changes and the degree of tissue damage as well as the severity of ischemia [7].

## Materials and methods

### Animal care

Experiments were carried out in accordance with the German Animal Welfare Legislation and were approved by the local institutional animal care and research advisory committee and permitted by the local government of Lower Saxony, Germany (AZ 10AO29). The study was performed at the experimental trauma surgery laboratory of Hannover Medical School (MHH).

Experiments were conducted in an operating room at the animal research facility. One hundred twelve male C57BL/6 N mice (Charles River, Germany) weighing 22 ± 3 g (gram) were used for the study. Twenty male mice were used in preliminary experiments to determine the weight needed for induction of chest trauma. All mice were handled at room temperature for 14 days before treatment, and all mice were age-matched (12 weeks old). We used only male C57BL/6 N mice for this primary study because gender of mice affects hormones and cell-mediated immune response [26, 27]. Cytokine expression also differs between male and female mice [28]. Further studies with female mice will be necessary.

Animals were maintained under standardized conditions in a controlled environment at 21 ± 2 °C (Celsius), with a relative humidity of 50% and artificial light (14 h light, 10 h dark). They received a commercial pellet diet (altromin 1320, Altromin, Lage, Germany) and water ad libitum. Analgesic treatment was administered to all animals in the form of metamizol-sodium (200 mg/kg (milligram/kilogram) body weight; Novalgin® Hoechst, Unterschleiβheim, Germany) throughout the study. Mice were injected subcutaneously under deep anesthesia prior to induction of the thorax trauma and after induction of the femur fracture. For postoperative analgesia, 0.8 mg/mL (milligram/milliliter) Novaminsulfon Lichtenstein 500 mg (Zentiva Pharma GmbH, Frankfurt am Main, Germany) was added to the drinking water for the first 3 days after trauma.

All surgical procedures were performed under deep anesthesia with isoflurane ((Minrad, Bethlehem, PA)) and local application of xylazine (16 mg/kg) (Rompun®, Bayer, Leverkusen, Germany). The mice were warmed to 36 °C using infrared heat lamps after the surgical procedures were complete. Wound closure was performed before recovery from the anesthesia.

For quantification of activity as a measure of clinical status after trauma, a scoring system was used [18].

### Group distribution and experimental procedures

Animals were randomly assigned to one of three groups. In the first group, mice received an isolated femur shaft fracture after stabilization with a pin (Fx). In the second group, mice received a combined intramedullary femoral fracture stabilization and blunt thoracic trauma (group TTFx). In the third group, mice underwent a control operation with intramedullary pin implantation in an intact femur without fracture (control).

Group Fx and TTFx were divided into six subgroups (*n* = 8) depending on the time point of sacrifice: 0 h, 6 h, 12 h, 24 h, 3 d and 7 d. The control group was sacrificed at 0 h (*n* = 8).

One hundred twelve multiple trauma, femur fracture and control mice were tested (48 multiple trauma and 48 isolated femur fracture). The control group consisted of 16 mice (16,6% (percent)) that underwent an operation (femur stabilization) in the absence of fracture or chest trauma (Figure 1c). Experiments were undertaken by three different surgeons. There were no significant differences with regard to moribund animals (surgeon 1: 6 moribund mice, surgeon 2: 4 moribund mice, surgeon 3: 6 moribund mice).

### Blunt thoracic trauma

After induction of the femur fracture, a blunt thoracic trauma was induced by a modified version of a previously described model for rats of bilateral lung contusion in the TTFx group [19, 29]. The method has been previously described as reproducible.

Blunt thoracic trauma was induced in anesthetized mice by dropping a hollow aluminum cylindrical weight (300 g) from a height of 55 cm (centimeter) through a vertical stainless steel tube onto a Lexon platform resting on the chest (Figure 1a, b). The impact energy E (1.617 J (Joule)) of the falling weight was calculated using the eq. E = m x g x h, where m = mass of aluminum weight (in kilograms), g = gravitational acceleration (9.8 ms^− 2^ (milliseconds^− 2^)) and h = height of weight above the Lexon platform (in meters). The calculations assumed that all the potential energy of the weight was transferred to the animal, neglecting frictional dissipation. The platform was suspended on Teflon guides to minimize friction and facilitate energy transfer to the anesthetized animal. The shield was reproducibly placed entirely over the chest without intrusion onto the abdomen.

The experiments were performed by three different autonomous surgeons. All data were examined by a statistician. The cause of death was determined during organ removal immediately after death.

### Femur fracture

The experimental design of the multiple trauma model is based on a two-hit model. The first hit consists of a closed femur fracture on the right side as described by Bonnarens [19]. In brief, under deep anesthesia with isoflurane, a 20 gauge needle was first inserted into the canal of the mouse femur as an intramedullary pin (Figure 1c). After primary wound closure, a standardized femur fracture was induced in both groups using a blunt guillotine device weighing 500 g (0.784 J) after primary stabilization. This procedure resulted in an A-type femoral fracture combined with a moderate soft-tissue injury. The type of fracture (A fracture, AO classification) was controlled after sacrifice.

### Body weight, activity and body temperature

The body weight, body temperature and activity of mice were measured in all groups before trauma and after trauma before sacrifice.

### Assessment of blood parameters

Samples for the control group were collected using the retrobulbar technique during the preliminary test. Posttraumatic control was performed by heart puncture. By using 2-mL (milliliter) syringes (Pico50, Radiometer Medical, Brønshøj, Denmark) containing 80 IU electrolyte-balanced heparin, blood samples (0.7 ml) for blood gas analysis and assessment of marker enzyme activities were collected from heart. The animals were sacrificed by heart puncture under deep anesthesia. The hemoglobin concentration, hematocrit and metabolic parameters (lactate, glucose), and osmolality were assessed using a blood gas analyzer (ABL 715, Radiometer, Copenhagen, Denmark).

### Specimens

Animals were sacrificed immediately after trauma, after 6 h, 12 h, 24 h, 3 d and 7 d to obtain samples for histologic examination. Tissue samples from lung were collected and stored at − 20 °C until processed.

### Histology

Tissue samples were embedded in paraffin. Sections (5 μm (micrometer)) were obtained from the central portion of the lung with a sliding microtome (HM 430; Microm International), placed on Superfrost Plus microscope slides (Thermo Scientific) and incubated overnight at 60 °C. The sections were routinely stained with hematoxylin and eosin (H&E). Safranin O staining was carried out for 6 min using a 0.1% aqueous solution at pH 3.0.

### Micro computed tomography

Chest trauma of multiple trauma mice was assessed by micro computed tomography (μCT).

The CT scan was performed at the Molecular Imaging North Competence Center (Am Botanischen Garten 14, 24118 Kiel). Micro computed tomography in Kiel has been applied previously to mice in several studies [30, 31]. Two radiologists planed every scan.

The total scan time was approximately 14 min. Scanning of mice lungs has been described previously [32]. The lungs of mice, which were killed immediately after trauma, were scanned using a Novotec MicroScope (Novotec Medical GmbH, Pforzheim) at an isotropic nominal spatial resolution (voxel size) of 15–20 μm. Samples were transported on ice before the scanned lungs were positioned on a special platform to prevent artifacts. Image analysis was performed using ImageJ software.

### Harvesting procedure

Animals were sacrificed under deep anesthesia with isoflurane at 0 h, 6 h, 12 h, 24 h and 3 d after trauma induction. Heparinized blood was obtained via cardiac puncture. Blood was centrifuged at 2500×*g* for 5 min (minutes) at room temperature (Eppendorf 3200, Hamburg, Germany). After centrifugation, the plasma was transferred into a fresh tube, snap-frozen and stored at − 80 °C.

### Protein analysis of cytokines

To analyze concentrations of different cytokines, blood samples obtained by heart puncture of the mice were centrifuged for five minutes. The supernatant was removed and stored at 20 °C until processing. The concentrations of IL-1β, IL-6, IL-10, IL-12p70 and TNFα in plasma samples were analyzed using a Luminex assay according to standard protocols with LiquiChip200 (Qiagen). A Milliplex cytokine multiplex immunoassay kit (MPXHCYTO-60 K-01; Millipore) was used for protein detection.

### Statistics

Statistical analysis was performed using a standard software application (SPSS Inc., Chicago, IL, USA). Differences between the sham group and the other groups were evaluated using the Wilcoxon signed-rank test. For compromise of mice with an isolated fracture and mice with chest trauma and a femur fracture we used further the Mann-Whitney U test. Probability values less than 0.05 were considered statistically significant. The data are shown as box-and-whisker plot with median and interquartile range.

## Results

### Survival

Regarding the reproducibility of the model in the group of multiple trauma mice, 16 mice (33%) died after chest trauma because of hemorrhage and 32 mice (66%) survived.

### Weight and temperature

All mice that underwent chest trauma and femur fracture showed a tendency of reduced weight after 6 h. Representative the weight loss was shown for 5 mice with an initial weight of 25,62 g, 25,58 g, 26,54 g, 24,40 g and 24,83 g. After 6 h the weight was reduced to 24,06 g, 24,95 g, 25,39 g, 24,16 g and 23,17 g (Figure 2). After 3 days the weight had returned completely to baseline values. Temperature declined until 24 h after trauma (Figure 3). Representative temperature was shown for 5 mice before (38,7 °C, 37,6 °C, 37,8 °C, 37,2 °C, 38,7 °C) and 24 h after the trauma (37,2 °C, 36,8 °C, 37,6 °C, 37 °C, 37,5 °C). After 24 h weight and temperature returned to baseline values. There was no significant decrease in either temperature or weight after trauma.

### Hemoglobin

In a previous examination 5 mice of the same age and weight were punctured retrobulbar before trauma induction as a control to measure the baseline hemoglobin values. Compared to the control group (14,8 g/dl (gram/deciliter), 15,3 g/dl, 14,4 g/dl, 14 g/dl, 15,3 g/dl, 14,9 g/dl), hemoglobin declined after 6 h (13,7 g/dl, 13 g/dl, 13,5 g/dl, 11,7 g/dl, 13,7 g/dl, 12,2 g/dl) and 12 h (13,7 g/dl, 11,7 g/dl, 13,2 g/ dl, 14,7 g/dl, 12,5 g/dl, 14,1 g/dl) until 24 h after induction of chest trauma (14,4 g/dl, 13,4 g/dl, 13,2 g/dl, 12 g/dl, 12,3 g/dl, 13,7 g/dl) (Figure 4). Hemoglobin values had returned to baseline values 3 days after trauma. All tested mice showed comparable results.

### CT

Macroscopic and microscopic analyses showed that immediate death was caused by intrathoracic bleeding or heart contusion. CT scans were performed on mice that were killed immediately after chest trauma (Figure 5). Hemothorax and lung contusion could be observed on the thoracic CT. The injured mice had no rib fractures. All mice showed comparable results on CT scan.

### Histology

Histological sections were examined to assess the severity of pulmonary tissue injury in mice (Figure 6). HE staining was performed for lung samples of mice with chest trauma and an unoperated control group. At 24 h post-contusion, HE staining of lung samples showed thickening of the alveolar lining with ongoing leukocytic infiltration. All of the stained lungs showed comparable results.

### Inflammatory markers

Different cytokines were analyzed by multiplex immunoassay in plasma samples of mice with an isolated femur fracture and in mice with a chest trauma and femur fracture (Figure 7a, b, c, d, e). Mice with an isolated femur fracture showed a down-regulation of IL1β from 0 h (808,84 ± 190,93 pg/ml (picogram/milliliter)) to 12 h (492,81 ± 190,93 pg/ml) and an up-regulation of IL-6 from 0 h (39,32 ± 18,58) to 6 h (76,67 ± 16,40). IL1β and IL-6 were up-regulated from 0 h (IL1β 209,12 ± 166,51 pg/ml; IL-6 22,98 ± 9,59 pg/ml) to 6 h after multiple trauma (IL1β 829,40 ± 163,87 pg/ml; IL-6 99,88 ± 65,18 pg/ml). IL-10 expression was down-regulated from 0 h (2464,06 ± 894,49) to 12 h (1773,15 ± 742,4) in mice with an isolated fracture. In contrast, the maximum of IL-10 expression was reached at 12 h after multiple trauma (2519,12 ± 1782,87 pg/ml), whereas IL-10 expression was reduced directly after trauma (0 h 1085,22 ± 702,85 pg/ml) compared with the control group (1644,08 ± 1001,46 pg/ml).

After an isolated femur fracture, an up-regulation of IL-12p70 could be detected from 0 h (679,24 ± 578,52) to 6 h (1523,32 ± 480,26). Similar to mice with an isolated fracture, IL-12p70 was up-regulated and could be detected from 0 h (321,64 ± 294,74 pg/ml) to 6 h after multiple trauma (1671,78 ± 350,74 pg/ml).

A slight regulation of TNFα expression was observed after an isolated fracture, whereas TNFα expression declined immediately after multiple trauma (0 h 578,93 ± 232,48 pg/ml) compared with the control group (1241,5 ± 266,22 pg/ml). The baseline level of TNFα expression was recovered at 6 h after trauma (1288,76 ± 693,74 pg/ml). After multiple trauma all cytokines (IL1β, IL-6, IL-12p70, IL-10 and TNFα) were up-regulated (Figure 7a, b, c, d, e). Mice with multiple trauma showed a significant up-regulation for IL-6 compared to mice with an isolated fracture.

## Discussion

Blunt chest trauma represents one of the most common injuries in multiple trauma patients [33], while lung contusion is one of the most important factors contributing to the increased morbidity and mortality of multiple trauma patients [34]. Femoral fractures represent one of the most prevalent associated injuries in multiple trauma patients with blunt thoracic trauma [35]. The presence of long bone fractures causing respiratory deterioration and respiratory dysfunction may preclude orthopedic surgical intervention for several days.

In our study, we investigated a reproducible new multiple trauma mouse model using the combination of these two major injuries. The main questions of the present study may be summarized as follows:

- Why were mice chosen as our experimental animal?
- Why did we choose the combination of chest trauma and femur fracture?
- What are the influences of chest trauma and femur fractures?
- How is the immune response altered in terms of cytokine expression?

Mice are currently the experimental tool of choice for the majority of immunologists, and the study of their immune responses has offered tremendous insight into the functions of the human immune system [36]. Humans and mice share approximately 80% of their genes [37], and unlike large animal models, mice are technically easier to implement, have lower acquisition and housing costs and superior ethical acceptance and are available as knockout animals.

Traumatic brain injury, thoracic trauma, hemorrhagic shock and long bone fracture are the focus of most mural trauma models. All these models have advantages and disadvantages.

Some trauma models focus on an isolated organ or tissue injury, and some models focus on the combination of several severe injuries. To concentrate on a particular injury might be an advantage in some ways, but it does not replicate multiple trauma in humans [38].

Several trauma studies focus on traumatic brain injuries [39–41]. The knowledge about outcome rates after concomitant traumatic brain injuries may help prioritize the research in this regard. However traumatic brain injury models have the limitation of not reflecting exactly the clinical setting and posttraumatic intensive monitoring in humans [42].

Hemorrhagic shock ist the leading cause of morbidity and mortality in trauma patients [43]. In mouse models hemorrhagic shock can be induced volume controlled, pressure controlled or uncontrolled [44]. While volume-controlled hemorrhagic shock shows compensatory physiological mechanisms and is easy and less invasive to perform, it provides the disadvantage of an uncertain severity of hemorrhage [45]. Pressure-controlled hemorrhagic shock models are standardized and reproducible models that allow the analysis of severe shock states and the monitoring of physiological parameters; however, they show suppression of compensatory mechanisms. Uncontrolled hemorrhagic shock models represent the clinical situation but are less standardized [45]. The manipulation of a single variable such as volume, blood pressure and time may cause unpredictable, irreproducible results so that hemorrhagic shock models are difficult to compare [45].

Chest trauma in small animals can be induced by, for instance, a blast wave generator [46] or weight-induced bilateral lung contusion [19], which we used in our model. Blast injury is an important cause of trauma in military conflicts or terrorism, whereas weight-induced trauma imitates the trauma that occurs in traffic accidents [47].

A blast generator created laser induced stress waves and the intensity of the shock wave is flexible by varying the laser energy [47]. The trauma model of bilateral lung contusion induced by a focused external blunt chest trauma (Figure 1a, b) has the advantages of being specific in terms of lung contusion (Figures 5 and 6) and uses a method of impact induction, which is reproducible and highly relevant to the chest trauma that occurs in motor vehicle accidents [19].

Despite the high incidence of chest trauma, it seems that immunologic alterations following pulmonary contusion are insufficiently elucidated. Apart from thoracic injuries, long bone fractures are particularly critical and represent one of the most prevalent associated injuries in multiple trauma patients with blunt thoracic injuries [35, 48]. Tibia fracture models have the advantage of easier intramedullary access compared to the femur [49]. The diameter of the femur is relatively consistent and large compared with that of the tibia, which facilitates the use of larger implants and the bone is more thickly covered by muscle [49].

In an open femur fracture model the bone is exposed and fractured via osteotomy or by weakening the bone with several drill holes [50]. Open fracture models are stabilized by extramedullary fixation techniques like a locking plate or an external fixator [44]. Induction of an open fracture and extramedullary stabilization generates considerable soft tissue injury. External fixation has the disadvantage of high implant weight and the large variation in implant stiffness [51]. An external plate fixation may damage the periosteum and perfusion and nutrition [51]. In contrast to most of the intramedullary stabilization techniques the external stabilization provides rotation stability after fracture [52].

In a closed femur fracture model the fracture usually followed by placement of intramedullary screws, pins or locking nails [20, 51, 52]. Closed fracture models have the advantage of reduced risk of wound infection compared to an open osteotomy [20]. Several intramedullary stabilization systems are not stable against longitudinal and rotational deformations [49]. A locking nail offers higher stability compared to pin fixation but is not a rigid fixation technique and the operation time is longer which might be a relevant disadvantage in a multiple trauma model [49]. Rotation stability can be achieved by flattening the tip and the distal end of a needle or a pin [51]. In our trauma mouse model, femur fracture was stabilized by minimal invasive surgery before the fracture was induced. Prior to fracturing the femur a 20 gauge needle was inserted in the medullary cavity of the femur to maintain axial alignment during the fracture and avoid large displacements. We chose this method described by Bonnarens [20] because it offers accurate reduction, a reduced operation time, less costs and less blood loss than external fixation [50]. While stabilization is performed immediately before the induction of fracture, the second hit inflammatory reaction can also be prohibited similar to damage control. Nevertheless, a higher incidence of complications and higher mortality after fracture stabilization is always observed in the presence of severe thoracic injuries [53].

Although early fracture stabilization can minimize several pulmonary complications such as fat embolism [54], damage control surgery and the timing of optimal treatment in multiple trauma patients are still points of interest [55]. Damage control during femur fracture stabilization has been shown to be beneficial for preventing the second hit inflammatory reaction and is associated with decreased blood loss and decreased mortality and morbidity in trauma patients [56]. There is evidence that early fracture fixation reduces the incidence of fracture-related complications and improves fracture outcome.

The mortality rates of multiple trauma patients range from 7 to 45%, depending on the injury severity [57]. In multiple trauma patients, 20–25% of deaths are attributed to chest injury [58]. In our study, 16 mice (33%) died within the first 30 min after chest trauma, and 64 mice (66%) survived. The mortality rate in our trauma model was high compared to other studies [46, 47, 59] but most of the thoracic trauma models focus on an isolated thoracic injury [47, 48, 59]. Examining isolated organ injury may be of benefit; however, this does not accurately replicate human trauma, which often involves multiple organ systems [38].

Additionally, in some models only one side or a special part of the chest is affected [47, 59] whereas our model is a bilateral contusion model [18]. In preliminary test we determined a high impact energy to generate a severe chest trauma. The thoracic trauma was first described for rats (body weight 250–300 g) with an impact energy of 2,45 J [19]. The falling weight in our study had an impact energy of 1,617 J, and the mice had a body weight of 22 ± 3 g. The impact energy we choose might be high in relation to the small mural chest.

The animals that did not survive died immediately after the chest trauma due to intrathoracic hemorrhage, which was confirmed by CT scan and removal of the organs. Interestingly, no rib fractures were found in our study or mentioned in previous evaluations [60, 61]. The murine chest exhibits high flexibility because of the costal dorso-ventral joints, which are not present in the human thorax [62, 63].

In humans, the metabolic response to severe injury results in hypothermia and weight loss. Similar to clinical conditions, the mice displayed a decrease in body temperature, weight loss and blood loss after multiple trauma during the first 24 h after trauma (Figures 2, 3 and 4).

Accidental hypothermia is a serious problem in multiple trauma patients because of the negative pathophysiological effects [64]. Early rewarming appears to be essential for the treatment of hypothermic trauma patients. In our study, the mice were placed under an incubator lamp for the first 12 h after induction of trauma, but body temperature did not recover until 24 h post-trauma potentially because only external warming was applied without the donation of warm infusions or a blood supply, which is normally administered to trauma patients.

The posttraumatic inflammatory reaction in humans and mice is essentially regulated by cytokine expression [65, 66]. The magnitude of cytokine expression is regulated by the trauma severity in humans [67]. TNFα, IL-6, IL-1β and IL-10 correlate with the systemic inflammatory response and injury severity [68, 69], and therefore we focused on these mediators in mice. Multiple trauma patients with severe damage or limited lung function exhibit significantly higher cytokine patterns in the early post-injury phase, with elevations of TNFα, IL-6, IL-10 and IL-1β compared with other trauma patients [70]. We detected an increase in TNFα, IL-6 and IL-1β in the plasma samples from multiple trauma mice.

We focused on IL-12, which is produced at high levels by monocytes and macrophages. Blunt chest trauma induces mediator-dependent monocyte migration to the lung [71], and high expression of IL-12 can be detected in the monocytes of trauma patients [72]. An increase of IL-12p70 could also be detected in multiple trauma mice.

In comparison to humans, a correlation between cytokine expression and the severity of trauma could also be detected in our trauma model. Mice with a single fracture generally showed reduced cytokine expression compared with multiple trauma mice.

TNFα, IL-6 and IL-1β are rapidly acting cytokines in humans, and peak levels can be detected within 24 h after trauma [73] since elevated levels can be detected after few minutes in humans [53]. Similar results were obtained for mice after trauma, with an increase observed at 24 h. Our results using the murine trauma model were also consistent with the results of an isolated blast wave trauma model or burn injury model [46]. In summary, mice seems to show comparable cytokine expression patterns to human trauma patients.

## Conclusion

We have established a new multiple trauma mouse model that better recapitulates the immunological response of severely injured patients. Despite clear differences between humans and animals, animal studies are necessary to gain further insight into the physiological mechanisms underlying multiple trauma.

This trauma model will be extremely helpful to answer outstanding questions concerning whether cytokine blockade, which is available in the clinic, is helpful for the treatment of trauma patients. Such studies can be further complemented by the evaluation of genetically modified mice that lack particular cytokines, in terms of the course of multiple traumatic situations. These studies will eventually lead to better therapeutic approaches to this life-threatening condition. Specifically, it is even more important to develop new animal models with the most frequently encountered injuries for further medical improvement, necessitating further studies.

## Figure Legends

Figure 1 **a**, **b** Apparatus used for the multiple trauma mouse model. A cylindrical weight of 500 g was dropped through a tube onto a plunger in contact with the chest of an anesthetized mouse. Mice were placed on a cross on a platform of acrylic glass immediately below the plunger. The plunger allowed induction of a bilateral pulmonary contusion. **c** Induction of femur fracture. Before a closed femur fracture was induced, the femur was stabilized using a cannula (Sterican 0.55 × 25 LILA lI) from the knee joint to the femoral neck. The method used to induce femur fracture was first described by Bonnarens. After primary wound closure, a standardized femur fracture was induced using a blunt guillotine device with a weight of 500 g (0.784 J)

Figure 2 Weight of the mice after multiple trauma. The mouse numbers are equal to the order of the data. The weight of mice was determined before multiple trauma and before each mouse was sacrificed. A decrease in weight was observed during the first 24 h following trauma (shown for 6 h after trauma)

Figure 3 Body temperature of the mice after multiple trauma. The mouse mice are equal to the order of the data. The body temperature (°C) of mice was measured before multiple trauma was induced and before each mouse was sacrificed. A drop in temperature was observed during the first 24 h after trauma (shown for 6 h after trauma)

Figure 4 Hemoglobin measurements in mice after multiple trauma. The hemoglobin level in mice without trauma was compared to hemoglobin level in mice after multiple trauma. Five mice without trauma underwent retrobulbar puncture to measure hemoglobin as a control. Mice that underwent trauma were sacrificed by heart puncture at 6 h, 12 h, 24 h, 3 d, 7 d, 14 d, and 28 d after trauma. Data are shown for 5 mice each at 6, 12, and 24 h after trauma. Blood samples were analyzed immediately after puncture using a Radiometer ABL 700. Hemoglobin levels decreased up to 24 h

Figure 5 CT scan of chest trauma. Computerized tomography was performed for 5 mice that died immediately after chest trauma. The CT scans revealed intrathoracic bleeding and hemopneumothorax. Scans were performed in the Molecular Imaging North Competence Center, CAU Kiel

Figure 6 Microscopic evaluation of histological changes in the lung of mice after pulmonary contusion. Mice subjected to chest injury showed extensive intra-alveolar and intrabronchial hemorrhaging with consecutive atelectasis, while sham mice did not show such pulmonary changes

FIgure 7 **a**, **b**, **c**, **d**, **e** Cytokine analysis assessed by the Luminex assay. Serum samples of mice with an isolated femur fracture (Fx) and mice with chest trauma and femur fracture (TTFx) were analysed by multiplex immunoassay. All Data are shown as box-and-whisker plot with median and interquartile range. Concentration of different cytokines (IL-1β, IL-6, IL-10, IL-12p70 and TNFα) was measured in different groups of mice 0 h, 6 h, 12 h, 3 d and 7 d (*n* = 8) after trauma. Figures show regulation of IL-1α, IL-6, IL-10, IL-12p70 and TNFα after an isolated femur fracture from 0 h to 7 d after trauma and cytokine regulation after induction of chest trauma and femur fracture from 0 h to 7 d after trauma. Cytokine expression was compared respectively to Sham group

## Abbreviations

%: Percent
°C: Celsius
ALI: Acute lung injury
ARDS: Acute respiratory distress syndrome
cm: Centimeter
d: Day
dl: Deciliter
E: Impact energy
Fx group: Fracture group
g: Gram
g: Gravitational acceleration
h: Height of weight above the Lexon platform (in meters)
h: Hour
H&E-stained: Haematoxylin and Eosin-stained
IL: Interleukin
J: Joule
Kg: Kilogram
m: Mass of aluminum weight
mg: Milligram
min: Minutes
ml: Milliliter
MODS: Multi organ dysfunction syndrome
ms: Millisecond
pg: Picogram
TNF-α: Tumor necrosis factor- α
TTFx group: Thorax trauma fracture
μCT: Micro computed tomography
μm: Micrometer
